# Multidisciplinary strategies to reduce radiotherapy-induced cardiotoxicity in breast cancer: surgical and technological innovations

**DOI:** 10.3389/fonc.2025.1647080

**Published:** 2025-08-13

**Authors:** Kai Lu, Zhenhua Sun, Yide Yi

**Affiliations:** ^1^ Department of Thyroid and Breast Surgery, Affiliated Hospital of Jiangsu University, Zhenjiang, China; ^2^ Institute of Medical Imaging and Artificial Intelligence of Jiangsu University, Affiliated Hospital of Jiangsu University, Zhenjiang, China

**Keywords:** breast cancer, radiotherapy, cardiotoxicity, cardiac protection, radiotherapy-induced cardiotoxicity

## Abstract

Radiotherapy remains essential in breast cancer management, yet its long-term cardiotoxicity, driven primarily by radiation-induced myocardial fibrosis, threatens survivorship, particularly in left-sided tumors. Surgical refinements, including breast-conserving surgery with sentinel lymph node biopsy and total mastectomy, effectively reduce radiation fields and cardiac exposure. Intraoperative radiotherapy with lead shielding markedly lowers left anterior descending artery dose from 5.2 Gy to 0.07 Gy. Technological advances—such as deep-inhalation breath-hold, proton therapy exploiting the Bragg peak, and intensity-modulated radiotherapy, further optimize cardiac sparing while preserving oncologic efficacy. Integrating intraoperative image guidance, pharmacological cardioprotection, and AI-assisted planning facilitates precise dose delivery tailored to individual anatomy and risk. This review synthesizes multidisciplinary strategies to mitigate cardiac injury through surgical and technological innovation, underscoring a paradigm shift toward organ-sparing precision radiotherapy. Future directions include the application of degradable shielding materials, senescence-targeted therapies, and predictive modeling to balance therapeutic efficacy with long-term cardiovascular safety in breast cancer care.

## 1 Introduction

Breast cancer remains the most prevalent malignancy among females worldwide, accounting for approximately 25% of all female cancers, with an estimated 2.3 million new cases diagnosed annually (1). Radiotherapy serves as a cornerstone in the multidisciplinary management of breast cancer, employed in 50-60% of cases to reduce local recurrence and improve survival (2). However, the therapeutic benefits of radiotherapy are counterbalanced by its potential cardiotoxicity, with 10-30% of survivors developing cardiovascular complications within a decade post-treatment. Left-sided breast irradiation poses particularly significant risks, conferring a 2.5-fold increased incidence of major coronary events compared to right-sided treatment (3, 4). The underlying pathophysiology involves radiation-induced myocardial fibrosis (RIMF), characterized by excessive collagen deposition (predominantly type I) and progressive cardiac dysfunction, which develops in 20-80% of exposed patients and substantially compromises long-term survivorship.

Surgical approaches play a pivotal role in modulating cardiac risk profiles. Mastectomy reduces cardiac radiation exposure by over 70% compared to breast-conserving surgery (5, 6), while advancements in tumor margin delineation and respiratory gating techniques enhance radiotherapy precision (7). As integral members of multidisciplinary teams, surgeons contribute crucially to preoperative risk assessment and postoperative cardiac monitoring (8, 9), optimizing both oncological control and cardioprotection. Emerging strategies such as intraoperative radiotherapy, proton therapy, and AI-enhanced planning, further refine cardiac sparing through precision dose delivery. This review synthesizes contemporary evidence on multidisciplinary approaches to mitigate radiotherapy-induced cardiotoxicity, highlighting innovations in surgical technique, radiotherapy technology, and predictive modeling to balance therapeutic efficacy with long-term cardiovascular safety in breast cancer care.

## 2 Mechanisms of radiotherapy-induced cardiotoxicity in breast cancer

### 2.1 Elevated collagen type I/III ratio in the RIMF process

Radiotherapy-induced cardiotoxicity in breast cancer primarily stems from ionizing radiation damaging cardiomyocytes and microvasculature, initiating oxidative stress and chronic inflammation. Under physiological conditions, myocardial extracellular matrix contains 3–5% interstitial collagen, predominantly type I with lesser type III (10). Type I collagen confers rigidity, while type III provides elasticity. Exceeding 5% collagen content with an elevated type I/III ratio increases myocardial stiffness, impairing compliance and diastolic function (11). Experimental and clinical evidence demonstrates that reducing type I collagen synthesis and lowering this ratio improves diastolic performance (12, 13), highlighting its therapeutic relevance in RIMF. Collagen turnover is modulated by multifactorial pathways. Radiation-induced cardiomyocyte and endothelial damage triggers TGF-β-mediated fibroblast activation, enhancing collagen deposition (14, 15). Damaged endothelium further amplifies fibrogenesis via leukocyte recruitment (16). The resultant pro-fibrotic milieu drives myeloid/endothelial-to-fibroblast differentiation, exacerbating collagen accumulation (13). Fibroblasts, the primary collagen-producing effectors in RIMF, disproportionately synthesize type I over type III collagen, elevating the I/III ratio. Targeting fibroblast activation attenuates this imbalance and temporarily ameliorates cardiac dysfunction (17). Nevertheless, persistent fibrosis progression implies additional regulators of collagen ratios remain unidentified. Therapeutically, senolytic agents (dasatinib + quercetin) have shown promise in clearing senescent fibroblasts and reducing fibrosis in animal models of radiation-induced injury (18). A pilot study is evaluating senolytics in cancer survivors with cardiac dysfunction, potentially offering a translatable strategy to mitigate RIM (19).

### 2.2 Senescent fibroblasts play a central role in the elevated collagen type I/III ratio

**Figure 1:**
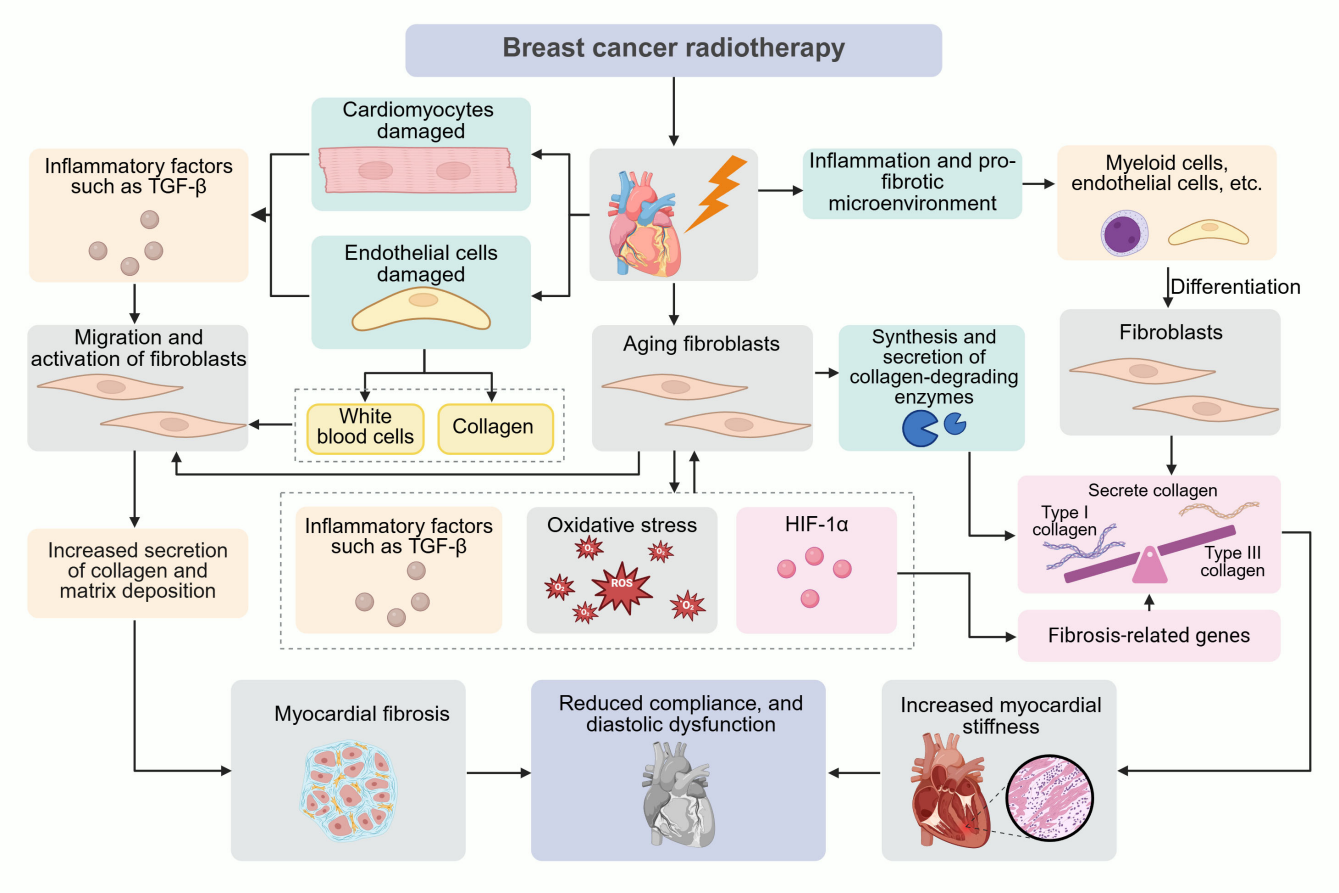
Pathophysiological mechanisms of radiotherapy-induced cardiotoxicity in breast cancer. This schematic integrates molecular and cellular events driving radiation-induced myocardial fibrosis: Ionizing radiation directly damages cardiomyocytes by triggering TGF-β release and endothelial cells via promoting leukocyte infiltration. Fibroblast activation amplifies collagen I/III deposition, increasing myocardial stiffness and impairing diastolic function. Senescent fibroblasts stabilize HIF-1α via ROS/TGF-β, upregulating profibrotic genes and collagenolytic enzymes that preferentially degrade fragile type III collagen. Myeloid/endothelial-to-fibroblast differentiation exacerbates fibrosis through collagen-enriched microenvironment formation.

Radiation-induced fibrotic lesions are predominantly composed of fibroblasts, whose persistent senescence accelerates disease progression despite potential early protective effects (20). In radiation-induced pulmonary fibrosis, fibroblast senescence severity directly correlates with fibrotic advancement (21). These cells mediate fibrosis through their senescence-associated secretory phenotype (SASP), establishing autocrine/paracrine feedforward loops via TGF-β and ROS that both expand senescent populations and recruit additional fibroblasts (22). The SASP induces a collagen imbalance through preferential degradation of structurally vulnerable type III collagen by senescent fibroblast-derived proteases, and oxygen-independent HIF-1α stabilization via SASP-generated ROS, which upregulates pro-fibrotic genes (23, 24). Paradoxically, while senescent fibroblasts exhibit diminished overall collagen production capacity, their selective enzymatic activity creates a collagen I-enriched extracellular matrix that characterizes progressive fibrosis (22) (**Figure 1**).

## 3 Optimization of surgical treatment and radiotherapy techniques

### 3.1 Impact of surgical modalities on the need for radiotherapy

**Figure 2:**
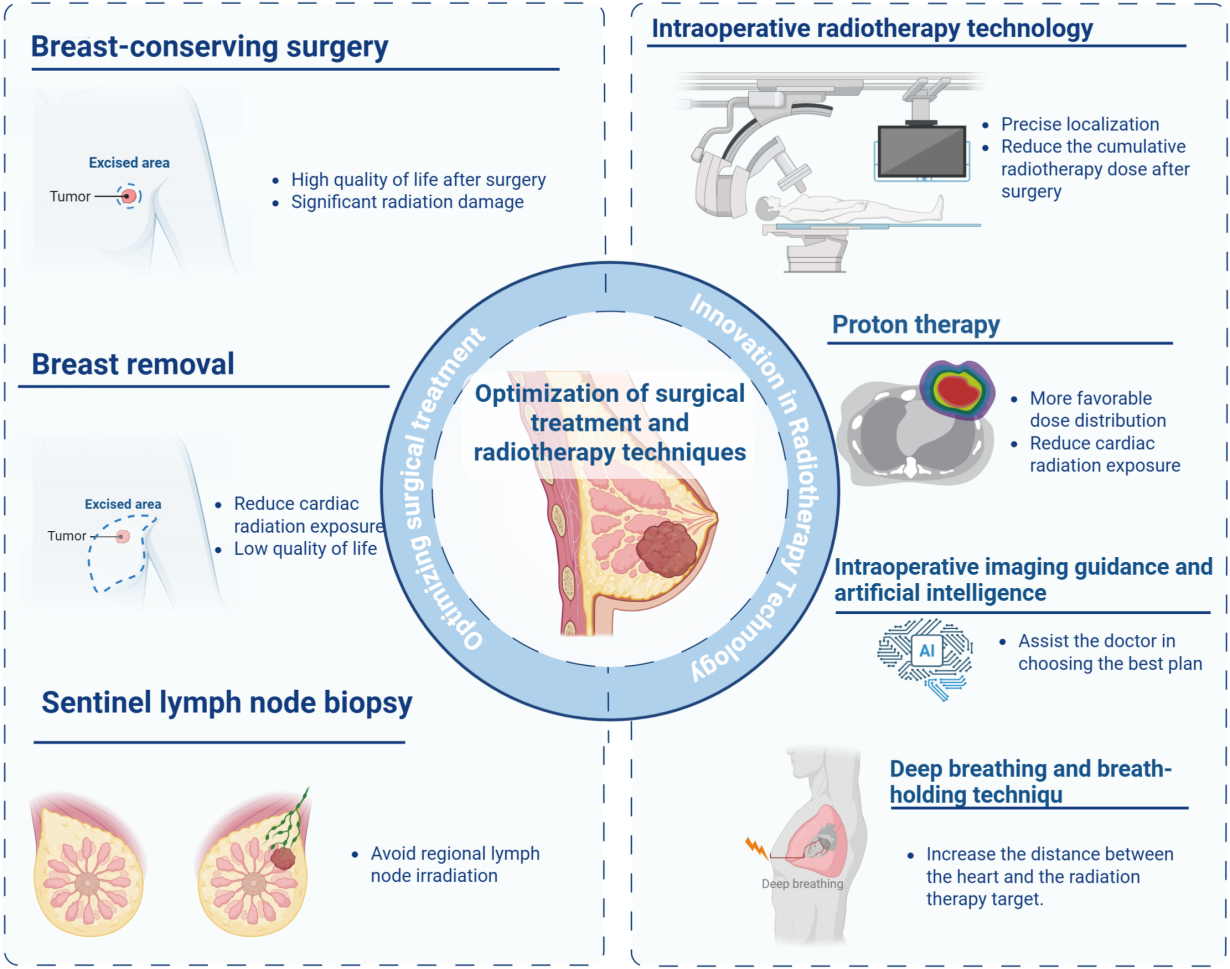
Surgical and radiotherapeutic strategies for cardiac risk reduction. Schematic representation of multidisciplinary strategies to reduce cardiac toxicity in breast cancer. Breast-conserving surgery preserves quality of life but increases cardiac radiation exposure. Total mastectomy reduces cardiac radiation dose at the cost of quality of life. Intraoperative radiotherapy enables precise tumor-bed targeting with reduced cumulative radiation dose. Proton therapy achieves favorable dose distribution to minimize cardiac exposure. Sentinel lymph node biopsy avoids regional lymph node irradiation. Intraoperative imaging guidance with artificial intelligence assists clinicians in optimal treatment planning, while the deep inspiration breath-hold technique increases heart-target distance during radiation delivery.

Breast cancer surgical approaches directly influence both oncological outcomes and subsequent radiotherapy needs, thereby modulating cardiotoxicity risks (**Figure 2**). Optimal patient selection is critical and should integrate tumor biology such as hormone receptor status and HER2 amplification, anatomical factors including tumor location relative to cardiac structures, and relevant comorbidities such as pre-existing cardiovascular disease and impaired pulmonary function (25). Modern surgical decision-making has transitioned from radical resection to a balanced approach incorporating organ preservation and cardiac risk mitigation, particularly for left-sided tumors. This paradigm shift emphasizes surgical strategy optimization to either eliminate or reduce radiotherapy exposure while maintaining oncological efficacy.

#### 3.1.1 Breast-conserving surgery increases cardiotoxic events in breast cancer

Breast-conserving surgery (BCS), while preserving breast aesthetics and improving quality of life, requires whole-breast irradiation (WBI), which elevates cardiac radiation exposure—particularly for left-sided tumors. Optimal patient selection should favor those with lower cardiac risk profiles, including small tumor size (≤3 cm), peripheral tumor location, unifocal disease, and minimal cardiovascular comorbidities (26). A Swedish-Danish case-control study demonstrated that mean cardiac radiation doses ranged from 0.03 to 27.72 Gy, with a linear 7.4% increase in major coronary events per Gy, beginning within five years post-radiation and persisting for decades without a safe threshold (8). For patients with high cardiovascular risk, including diabetes, prior coronary artery disease, alternative strategies such as mastectomy or proton therapy may be preferable (27). Emerging evidence supports selective omission of radiotherapy in low-risk subgroups. Women ≥65–70 years with stage I hormone receptor-positive tumors on endocrine therapy exhibit minimal recurrence risk without irradiation (28). Similarly, the IDEA trial (n=200, ages 50–69, pT1N0 unifocal disease) reported 3.3–3.6% ipsilateral breast event rates without radiotherapy (28, 29). These findings underscore the importance of individualized decision-making, balancing oncologic efficacy, cardiac safety, and patient preferences (30).

#### 3.1.2 Total mastectomy and indirect cardiac protection

Total mastectomy significantly reduces radiotherapy requirements by removing target breast tissue, thereby substantially decreasing cardiac radiation exposure. This approach proves particularly beneficial for patients with tumors >5 cm, centrally or medially located lesions near the sternum, multicentric disease, or genetic predisposition such as BRCA1/2 mutations, where radiotherapy indications are more compelling (31). Current Chinese Anti-Cancer Society guidelines restrict postmastectomy radiotherapy (PMRT) to T1/T2 tumors ≥5 cm or those with ≥1–4 lymph node metastases. A recent international phase II trial comparing conventional (50 Gy/25 fractions) versus hypofractionated proton PMRT (40.05 Gy/15 fractions) demonstrated excellent cardiac protection: mean cardiac doses were minimal (0.54 Gy vs 0.49 Gy), with median maximal left anterior descending artery doses of ~9 Gy. Both protocols maintained cardiac V25 significantly below photon radiotherapy benchmarks. Three-year outcomes showed 89.4-92.4% disease-free survival, zero local recurrences, and only 7 distant metastases, with no reported symptomatic cardiac events (32). For young left-sided tumor patients with extended life expectancy, total mastectomy with immediate reconstruction offers a viable cardioprotective option, though quality-of-life considerations remain crucial. In cases of small, peripherally located tumors, accelerated partial breast irradiation (APBI) with intraoperative tumor-bed marking presents an effective alternative, demonstrating comparable long-term control to whole-breast irradiation while achieving superior toxicity profiles and cosmetic outcomes (33).

#### 3.1.3 Sentinel lymph node biopsy optimizes postoperative radiotherapy strategies in breast cancer

Sentinel lymph node biopsy (SLNB) has revolutionized postoperative radiotherapy planning by enabling precise nodal staging while minimizing morbidity. Compared to axillary lymph node dissection (ALND), SLNB facilitates individualized radiotherapy approaches: node-negative patients can avoid regional nodal irradiation, significantly reducing cardiac exposure, while those with micrometastases (≤2 mm) benefit from modern techniques like deep-inspiration breath-hold (DIBH) and intensity-modulated radiotherapy (IMRT) to spare cardiac tissue. The therapeutic equivalence of SLNB-based strategies is well established. A multinational trial demonstrated comparable 5-year axillary recurrence rates between ALND and axillary radiotherapy (ART), with ART significantly reducing lymphedema risk (34). These findings were reinforced by the 2024 SENOMAC trial, which showed equivalent 5-year recurrence-free survival (89.7% vs. 88.7%) for SLNB alone versus ALND in patients with 1–2 nodal metastases (35). For early-stage disease, intraoperative radiotherapy further minimizes cardiotoxicity through precise tumor bed targeting and real-time cardiac monitoring during IMRT planning. However, caution remains regarding overtreatment reduction; while ALND fails to improve outcomes and increases lymphedema risk (36), radiotherapy omission after breast-conserving surgery elevates 15-year local recurrence from 10% to 30% (36). This underscores the importance of balanced, multidisciplinary decision-making to optimize both oncologic control and cardioprotection.

### 3.2 Intraoperative radiotherapy techniques

Intraoperative radiotherapy (IORT) delivers a single high-dose radiation to the tumor bed during breast-conserving surgery, precisely targeting areas at highest risk for residual disease (37). This approach offers distinct advantages over conventional external beam radiotherapy (EBRT), including reduced cardiac radiation exposure (mean dose 0.03 Gy vs 4.3 Gy) and preservation of breast tissue integrity (38, 39). However, its clinical implementation remains limited by stringent patient selection criteria and specialized infrastructure requirements (40). Modern IORT techniques employ 20–21 Gy electron beams or low-energy X-rays, achieving direct tumoricidal effects while minimizing cardiac exposure through lead shielding. The TARGIT-A trial demonstrated comparable oncological outcomes between IORT and EBRT (local recurrence 3.3% vs 1.3%), with significantly reduced cardiac mortality (0.9% vs 2.1%) (41). Dosimetric analyses confirmed superior organ protection, with left anterior descending artery exposure reduced from 5.2 Gy to 0.07 Gy (42). Critical contraindications include tumors >5 cm, multifocal disease, central tumor location, and positive margins - all mandating supplemental EBRT (43, 44). The procedure requires meticulous multidisciplinary coordination, extending operative time by 30–45 minutes for applicator placement and organ shielding (45-47). Technical refinements, including intraoperative clip placement and frozen section margin assessment, have improved target accuracy and reduced reoperation rates (43, 46, 48). Long-term follow-up from major trials confirms IORT’s non-inferiority, with TARGIT-A and ELIOT reporting 3.3% and 4.4% local recurrence rates respectively (47). Notably, outcomes vary by molecular subtype, with HR+/HER2- tumors demonstrating >90% 5-year metastasis-free survival (49). Current limitations include restricted availability in non-tertiary centers and insufficient long-term cardiotoxicity data, particularly for HER2-positive disease (44). Emerging innovations encompass portable delivery systems and AI-enhanced dose prediction models using 4D-CT anatomical mapping (50, 51).

## 4 Technological innovations in radiotherapy

### 4.1 Deep inhalation breath holding technique

The Deep Inhalation Breath Hold (DIBH) technique has emerged as a cornerstone in minimizing cardiac radiation exposure during left-sided breast cancer radiotherapy. By instructing patients to take a deep breath and hold it, DIBH exploits the downward displacement of the diaphragm and anterior movement of the chest wall to increase the distance between the heart and radiation fields, especially shielding the left anterior descending (LAD) artery (52). Successful implementation requires rigorous patient selection: ideal candidates demonstrate adequate pulmonary function (FEV1 ≥70% predicted), cognitive capacity to follow commands, and absence of severe emphysema/pleural adhesions limiting diaphragmatic excursion (43, 44). A German study involving 130 patients receiving internal mammary lymph node radiotherapy after left-sided breast cancer demonstrated significantly lower cardiac radiation doses in the DIBH group compared to free breathing. The mean heart dose decreased from 2.2 Gy to 1.3 Gy, while the mean left ventricular dose declined. Notably, LAD exposure dropped substantially from 14.3 Gy to 4.1 Gy, with LAD V15–V40 reduced by nearly 100% (53). However, effective implementation of DIBH is heavily reliant on patient cooperation and multidisciplinary preparation. Standardized training protocols are critical and typically include three components. First, patients undergo pre-treatment coaching consisting of two to three sessions, each lasting approximately 15 minutes, incorporating visual feedback through spirometry or video monitoring. Second, patients are instructed to perform daily incentive spirometer exercises at home for one week to enhance respiratory control. Third, intraoperative markers, such as surgical clips placed at the tumor bed, are utilized to improve the reproducibility of breath-hold during radiotherapy. A Turkish study compared coached (cDIBH) and non-coached (ncDIBH) patient groups. Patients in the cDIBH group—who received nurse-led instruction and a training booklet a week prior—had significantly shorter setup times (181.56 vs. 280.44 seconds) despite comorbidities like older age and lung disease. While overall cardiac doses were lower in the cDIBH group, only the maximal LAD dose showed a statistically significant reduction (29.5 Gy vs. 36.5 Gy) (54) (**Figure 2**). These findings underscore that structured coaching enhances the clinical efficiency and cardioprotective benefits of DIBH. In left-sided breast cancer, where radiotherapy poses substantial cardiac risk, early and systematic patient engagement offers a pragmatic strategy to optimize therapeutic outcomes while minimizing long-term toxicity.

### 4.2 Proton therapy

Proton therapy (PT) has emerged as a superior radiotherapeutic modality for breast cancer compared to conventional photon-based approaches, owing to its unique physical properties and enhanced dosimetric advantages (55). The characteristic Bragg peak phenomenon enables precise dose deposition within the target volume while dramatically reducing exit dose, thereby offering unparalleled organ-at-risk sparing - particularly for cardiac structures in left-sided breast cancer cases (56). In 2021, the Breast Cancer Subcommittee of the International Federation for Ion Therapy established a clinical consensus that PT demonstrates significant dosimetric superiority over 3D-conformal radiation therapy (3D-CRT) and intensity-modulated radiotherapy (IMRT), achieving both improved target coverage and substantial reductions in radiation exposure to critical organs (57). Notably, for postmastectomy patients with high recurrence risk, PT maintains exceptional target coverage while limiting mean cardiac doses to <1 Gy, representing a potential paradigm shift in radiation oncology practice. This cardioprotective benefit has been quantitatively validated by a comprehensive systematic review confirming PT’s superiority in minimizing cardiac radiation exposure among all radiotherapy modalities for left-sided breast cancer (58). However, despite these clear clinical advantages, the widespread implementation of PT faces practical challenges, primarily related to limited facility availability and considerable cost constraints, which currently restrict its routine clinical application.

### 4.3 Intensity-modulated radiotherapy

Intensity-modulated radiotherapy (IMRT) and volumetric modulated arc therapy (VMAT) represent advanced radiation techniques that have revolutionized cardiac sparing in breast cancer treatment through sophisticated multi-angle dose delivery. Compared to conventional 3D-conformal radiotherapy (3D-CRT), IMRT demonstrates superior dosimetric outcomes, reducing cardiac V25 exposure by 40.6% while simultaneously improving target volume dose homogeneity by 15-20% (59). However, this enhanced conformality comes with a paradoxical increase in low-dose radiation exposure to adjacent tissues, potentially elevating secondary malignancy risks. Recent investigations have revealed complex cardiac dose relationships in IMRT applications. The landmark study by Coon et al. demonstrated that while IMRT successfully reduced cardiac V35 by 80.6% (3.6% to 0.7%) in left-sided breast cancer cases, it paradoxically increased both mean cardiac dose from 2.63 Gy to 4.04 Gy and V20 exposure (60). Emerging technologies offer adjunctive solutions. The 2025 breakthrough by Chinese researchers introduced a dual-modality imaging platform combining surface-enhanced Raman scattering (SERS) with bioluminescence detection. This system achieved 92.3% sensitivity in detecting subclinical triple-negative breast cancer metastases through bio-orthogonal labeling, enabling real-time therapeutic monitoring at radiation doses 30-40% below conventional requirements (61). In sum, while IMRT and VMAT offer improved conformality and cardiac protection, careful planning is required to mitigate unintended low-dose exposure and long-term risks.

### 4.4 Integration of intraoperative image guidance and artificial intelligence

The integration of intraoperative image guidance and artificial intelligence (AI) has revolutionized radiotherapy planning for breast cancer by enabling preoperative simulation of cardiac dose exposure based on surgical approaches. AI-driven dose prediction models leverage machine learning algorithms to optimize surgical decision-making by forecasting the impact of different procedural strategies on radiation exposure to critical organs. A landmark study by the Netherlands Cancer Institute, involving 1,228 breast cancer patients, demonstrated that AI-enhanced FDG-PET/CT, utilizing radiomic feature extraction with random forest classifiers, significantly improved the detection of subclinical nodal metastases (AUC = 0.92 vs. 0.78 for conventional imaging), leading to modifications in 23% of regional treatment plans (62). Furthermore, Magnetic Resonance-guided Radiation Therapy (MRgRT) has emerged as a transformative modality, offering superior soft-tissue contrast and high-resolution real-time imaging. This technology enables precise delineation of target volumes and dynamic tracking of anatomical changes during treatment, making it particularly advantageous for breast cancer cases involving substantial tissue deformation (63). Despite these advancements, several challenges persist. The efficacy of Deep Inspiration Breath Hold (DIBH) techniques, for instance, is contingent upon patient compliance, which may be suboptimal in elderly individuals or those with pulmonary or cognitive impairments, potentially compromising treatment precision (64, 65). Concurrently, research into novel shielding materials, such as lightweight bismuth-based composites, has shown promise in providing effective radiation protection while minimizing side effects compared to traditional lead-based shielding (66). Collectively, these innovations signify a paradigm shift in breast cancer radiotherapy—from conventional “dose compromise” strategies toward a precision-based “organ-sparing” approach, ultimately enhancing therapeutic outcomes while mitigating toxicity.

## 5 Conclusion

Breast cancer survivorship has improved significantly, yet radiotherapy-induced cardiotoxicity remains a critical challenge, particularly for left-sided tumors. This review synthesizes evidence on surgical and radiotherapeutic strategies to mitigate cardiac risks while maintaining oncologic efficacy. Key findings demonstrate that breast-conserving surgery, though beneficial for quality of life, necessitates whole-breast irradiation, increasing cardiac exposure by 7.4% per Gy with persistent coronary risks. Conversely, mastectomy and proton therapy reduce mean heart doses to <1 Gy, offering substantial cardioprotection. Sentinel lymph node biopsy and AI-driven adaptive radiotherapy refine precision, enabling personalized risk stratification.

However, challenges persist, such as proton therapy’s limited accessibility and IMRT’s low-dose bath effects. A multidisciplinary approach, integrating surgical optimization, advanced radiotherapy, and cardiac surveillance, is essential to balance survival gains with long-term cardiovascular health. Future research must prioritize pragmatic randomized trials comparing conventional versus emerging techniques, with composite endpoints integrating both oncologic control and cardiotoxic events. International consortia should harmonize cardiac dosimetric constraints using AI-derived dose-effect models, particularly for vulnerable substructures like the LAD ostium.

## References

[1] Kim J Harper A McCormack V Sung H Houssami N Morgan E. Global patterns and trends in breast cancer incidence and mortality across 185 countries. Nat Med. (2025) 31:1154–62. doi: 10.1038/s41591-025-03502-3, PMID: 39994475

[2] Bergom C Bradley JA Ng AK Samson P Robinson C Lopez-Mattei J. Past, present, and future of radiation-induced cardiotoxicity: refinements in targeting, surveillance, and risk stratification. JACC CardioOncol. (2021) 3:343–59. doi: 10.1016/j.jaccao.2021.06.007, PMID: 34604796 PMC8463722

[3] Wang B Wang H Zhang M Ji R Wei J Xin Y. Radiation-induced myocardial fibrosis: Mechanisms underlying its pathogenesis and therapeutic strategies. J Cell Mol Med. (2020) 24:7717–29. doi: 10.1111/jcmm.15479, PMID: 32536032 PMC7348163

[4] Lyon AR López-Fernández T Couch LS Asteggiano R Aznar MC Bergler-Klein J. 2022 ESC Guidelines on cardio-oncology developed in collaboration with the European Hematology Association (EHA), the European Society for Therapeutic Radiology and Oncology (ESTRO) and the International Cardio-Oncology Society (IC-OS): Developed by the task force on cardio-oncology of the European Society of Cardiology (ESC). Eur Heart J. (2022) 43:4229–361. doi: 10.1093/eurheartj/ehac244, PMID: 36017568

[5] Ghasemi K Vaseghi G Mansourian M. Pharmacological interventions for preventing anthracycline-induced clinical and subclinical cardiotoxicity: A network meta-analysis of metastatic breast cancer. J Oncol Pharm Pract. (2021) 27:414–27. doi: 10.1177/1078155220965674, PMID: 33081570

[6] Henry NL. Optimising therapy and avoiding overtreatment in breast cancer. Lancet Oncol. (2025) 26:2–3. doi: 10.1016/S1470-2045(24)00707-1, PMID: 39675377

[7] Zeng C Xiong W Li X Reyngold M Gewanter RM Cuaron JJ. Intrafraction tumor motion during deep inspiration breath hold pancreatic cancer treatment. J Appl Clin Med Phys. (2019) 20:37–43. doi: 10.1002/acm2.12577, PMID: 30933428 PMC6523018

[8] Darby SC Ewertz M McGale P Bennet AM Blom-Goldman U Brønnum D. Risk of ischemic heart disease in women after radiotherapy for breast cancer. N Engl J Med. (2013) 368:987–98. doi: 10.1056/NEJMoa1209825, PMID: 23484825

[9] Brain E Caillet P de Glas N Biganzoli L Cheng K Lago LD. HER2-targeted treatment for older patients with breast cancer: An expert position paper from the International Society of Geriatric Oncology. J Geriatr Oncol. (2019) 10:1003–13. doi: 10.1016/j.jgo.2019.06.004, PMID: 31235436

[10] Nagalingam RS Chattopadhyaya S Al-Hattab DS Cheung DYC Schwartz LY Jana S. Scleraxis and fibrosis in the pressure-overloaded heart. Eur Heart J. (2022) 43:4739–50. doi: 10.1093/eurheartj/ehac362, PMID: 36200607

[11] Singh D Rai V Agrawal DK. Regulation of collagen I and collagen III in tissue injury and regeneration. Cardiol Cardiovasc Med. (2023) 7:5–16. doi: 10.26502/fccm.92920302, PMID: 36776717 PMC9912297

[12] Mendieta G Ben-Aicha S Gutiérrez M Casani L Aržanauskaitė M Carreras F. Intravenous statin administration during myocardial infarction compared with oral post-infarct administration. J Am Coll Cardiol. (2020) 75:1386–402. doi: 10.1016/j.jacc.2020.01.042, PMID: 32216907

[13] Wei Y Sun Y Liu J Zhang G Qin X Xu S. Early detection of radiation-induced myocardial damage by [(18)F]AlF-NOTA-FAPI-04 PET/CT imaging. Eur J Nucl Med Mol Imaging. (2023) 50:453–64. doi: 10.1007/s00259-022-05962-y, PMID: 36121463

[14] Fan M Yang K Wang X Chen L Gill PS Ha T. Lactate promotes endothelial-to-mesenchymal transition via Snail1 lactylation after myocardial infarction. Sci Adv. (2023) 9:eadc9465. doi: 10.1126/sciadv.adc9465, PMID: 36735787 PMC9897666

[15] Tzahor E Dimmeler S. A coalition to heal-the impact of the cardiac microenvironment. Science. (2022) 377:eabm4443. doi: 10.1126/science.abm4443, PMID: 36048959

[16] Garlapati V Molitor M Michna T Harms GS Finger S Jung R. et al: Targeting myeloid cell coagulation signaling blocks MAP kinase/TGF-β1-driven fibrotic remodeling in ischemic heart failure. J Clin Invest. (2023) 133:e156436. doi: 10.1172/JCI156436, PMID: 36548062 PMC9927945

[17] Frangogiannis NG. Transforming growth factor-β in myocardial disease. Nat Rev Cardiol. (2022) 19:435–55. doi: 10.1038/s41569-021-00646-w, PMID: 34983937

[18] Kirkland JL Tchkonia T. Senolytic drugs: from discovery to translation. J Intern Med. (2020) 288:518–36. doi: 10.1111/joim.13141, PMID: 32686219 PMC7405395

[19] Seara FAC Kasai-Brunswick TH Nascimento JHM Campos-de-Carvalho AC. Anthracycline-induced cardiotoxicity and cell senescence: new therapeutic option? Cell Mol Life Sci. (2022) 79:568. doi: 10.1007/s00018-022-04605-7, PMID: 36287277 PMC11803035

[20] Luan Y Zhu X Jiao Y Liu H Huang Z Pei J. Cardiac cell senescence: molecular mechanisms, key proteins and therapeutic targets. Cell Death Discov. (2024) 10:78. doi: 10.1038/s41420-023-01792-5, PMID: 38355681 PMC10866973

[21] Guan R Yuan L Li J Wang J Li Z Cai Z. Bone morphogenetic protein 4 inhibits pulmonary fibrosis by modulating cellular senescence and mitophagy in lung fibroblasts. Eur Respir J. (2022) 60:2102307. doi: 10.1183/13993003.02307-2021, PMID: 35777761 PMC9808813

[22] López-Otín C Blasco MA Partridge L Serrano M Kroemer G. Hallmarks of aging: An expanding universe. Cell. (2023) 186:243–78. doi: 10.1016/j.cell.2022.11.001, PMID: 36599349

[23] Janbandhu V Tallapragada V Patrick R Li Y Abeygunawardena D Humphreys DT. Hif-1a suppresses ROS-induced proliferation of cardiac fibroblasts following myocardial infarction. Cell Stem Cell. (2022) 29:281–297.e212. doi: 10.1016/j.stem.2021.10.009, PMID: 34762860 PMC9021927

[24] Yang JH Hayano M Griffin PT Amorim JA Bonkowski MS Apostolides JK. Loss of epigenetic information as a cause of mammalian aging. Cell. (2023) 186:305–326.e327. doi: 10.1016/j.cell.2022.12.027, PMID: 36638792 PMC10166133

[25] Haque W Butler EB Teh BS. Personalized radiation therapy for breast cancer. Curr Oncol. (2024) 31:1588–99. doi: 10.3390/curroncol31030121, PMID: 38534954 PMC10969188

[26] Chua BH. Omission of radiation therapy post breast conserving surgery. Breast. (2024) 73:103670. doi: 10.1016/j.breast.2024.103670, PMID: 38211516 PMC10788792

[27] Qiao K Wei Y Tao C Zhu J Yuan S. Proton therapy for breast cancer: Reducing toxicity. Thorac Cancer. (2024) 15:2156–65. doi: 10.1111/1759-7714.15451, PMID: 39275876 PMC11496198

[28] Jayasekera J Schechter CB Sparano JA Jagsi R White J Chapman JW. Effects of radiotherapy in early-stage, low-recurrence risk, hormone-sensitive breast cancer. J Natl Cancer Inst. (2018) 110:1370–9. doi: 10.1093/jnci/djy128, PMID: 30239794 PMC6292790

[29] Jagsi R Griffith KA Harris EE Wright JL Recht A Taghian AG. Omission of radiotherapy after breast-conserving surgery for women with breast cancer with low clinical and genomic risk: 5-year outcomes of IDEA. J Clin Oncol. (2024) 42:390–8. doi: 10.1200/JCO.23.02270, PMID: 38060195 PMC11846025

[30] Vidya R Leff DR Green M McIntosh SA St John E Kirwan CC. Innovations for the future of breast surgery. Br J Surg. (2021) 108:908–16. doi: 10.1093/bjs/znab147, PMID: 34059874

[31] Ciabattoni A Gregucci F De Rose F Falivene S Fozza A Daidone A. AIRO breast cancer group best clinical practice 2022 update. Tumori. (2022) 108:1–144. doi: 10.1177/03008916221088885, PMID: 36112842

[32] Mutter RW Giri S Fruth BF Remmes NB Boughey JC Harless CA. Conventional versus hypofractionated postmastectomy proton radiotherapy in the USA (MC1631): a randomised phase 2 trial. Lancet Oncol. (2023) 24:1083–93. doi: 10.1016/S1470-2045(23)00388-1, PMID: 37696281 PMC10591844

[33] Icro M Marrazzo L Calogero S Isacco D Vieri S Gabriele S. Accelerated partial-breast irradiation compared with whole-breast irradiation for early breast cancer: long-term results of the randomized phase III APBI-IMRT-florence trial. J Clin Oncol. (2020) 38:4175–4183. doi: 10.1200/JCO.20.00650, PMID: 32840419

[34] Donker M van Tienhoven G Straver ME Meijnen P van de Velde CJ Mansel RE. Radiotherapy or surgery of the axilla after a positive sentinel node in breast cancer (EORTC 10981–22023 AMAROS): a randomised, multicentre, open-label, phase 3 non-inferiority trial. Lancet Oncol. (2014) 15:1303–10. doi: 10.1016/S1470-2045(14)70460-7, PMID: 25439688 PMC4291166

[35] de Boniface J Filtenborg Tvedskov T Rydén L Szulkin R Reimer T Kühn T. Omitting axillary dissection in breast cancer with sentinel-node metastases. N Engl J Med. (2024) 390:1163–75. doi: 10.1056/NEJMoa2313487, PMID: 38598571

[36] Clarke M Collins R Darby S Davies C Elphinstone P Evans V. Effects of radiotherapy and of differences in the extent of surgery for early breast cancer on local recurrence and 15-year survival: an overview of the randomised trials. Lancet. (2005) 366:2087–106. doi: 10.1016/S0140-6736(05)67887-7, PMID: 16360786

[37] Silverstein MJ Kim B Lin K Lloyd S Snyder L Khan S. Intraoperative radiation therapy (IORT) for breast cancer: the final analysis of a prospective cohort of 1828 cases. Ann Surg Oncol. (2025) 32:5563–5571. doi: 10.1245/s10434-025-17698-8, PMID: 40450167 PMC12222354

[38] Vaidya JS Vaidya UJ Baum M Bulsara MK Joseph D Tobias JS. Global adoption of single-shot targeted intraoperative radiotherapy (TARGIT-IORT) for breast cancer-better for patients, better for healthcare systems. Front Oncol. (2022) 12:786515. doi: 10.3389/fonc.2022.786515, PMID: 36033486 PMC9406153

[39] He J Chen S Ye L Sun Y Dai Y Song X. Intraoperative radiotherapy as a tumour-bed boost combined with whole breast irradiation versus conventional radiotherapy in patients with early-stage breast cancer: A systematic review and meta-analysis. Ann Surg Oncol. (2023) 30:8436–52. doi: 10.1245/s10434-023-13955-w, PMID: 37507556 PMC10625949

[40] Vinante L Vaidya JS Caroli A Mileto M Piccoli E Avanzo M. Real world clinical outcomes from targeted intraoperative radiotherapy (TARGIT-IORT) during lumpectomy for breast cancer: data from a large cohort at a national cancer institute. Front Oncol. (2024) 14:1424630. doi: 10.3389/fonc.2024.1424630, PMID: 39421443 PMC11484062

[41] Vaidya JS Wenz F Bulsara M Tobias JS Joseph DJ Keshtgar M. Risk-adapted targeted intraoperative radiotherapy versus whole-breast radiotherapy for breast cancer: 5-year results for local control and overall survival from the TARGIT-A randomised trial. Lancet. (2014) 383:603–13. doi: 10.1016/S0140-6736(13)61950-9, PMID: 24224997

[42] Tan W Wang X Qiu D Liu D Jia S Zeng F. Dosimetric comparison of intensity-modulated radiotherapy plans, with or without anterior myocardial territory and left ventricle as organs at risk, in early-stage left-sided breast cancer patients. Int J Radiat Oncol Biol Phys. (2011) 81:1544–51. doi: 10.1016/j.ijrobp.2010.09.028, PMID: 21470785

[43] Garcia MT Mota BS Cardoso N Martimbianco ALC Ricci MD Carvalho FM. Accuracy of frozen section in intraoperative margin assessment for breast-conserving surgery: A systematic review and meta-analysis. PloS One. (2021) 16:e0248768. doi: 10.1371/journal.pone.0248768, PMID: 33735315 PMC7971883

[44] Ciabattoni A Gregucci F Fastner G Cavuto S Spera A Drago S. IOERT versus external beam electrons for boost radiotherapy in stage I/II breast cancer: 10-year results of a phase III randomized study. Breast Cancer Res. (2021) 23:46. doi: 10.1186/s13058-021-01424-9, PMID: 33849606 PMC8045244

[45] Vaidya JS Bulsara M Baum M Wenz F Massarut S Pigorsch S. Long term survival and local control outcomes from single dose targeted intraoperative radiotherapy during lumpectomy (TARGIT-IORT) for early breast cancer: TARGIT-A randomised clinical trial. Bmj. (2020) 370:m2836. doi: 10.1136/bmj.m2836, PMID: 32816842 PMC7500441

[46] Ding Y Li J Wang W Wang S Fan T Xu M. Displacement of the lumpectomy cavity defined by surgical clips and seroma based on 4D-CT scan for external-beam partial breast irradiation after breast-conserving surgery: a comparative study. Br J Radiol. (2013) 86:20130416. doi: 10.1259/bjr.20130416, PMID: 23995875 PMC3798338

[47] Esposito E Anninga B Honey I Ross G Rainsbury D Laws S. Is IORT ready for roll-out? Ecancermedicalscience. (2015) 9:516. doi: 10.3332/ecancer.2015.516, PMID: 25793013 PMC4360616

[48] Dowling GP Hehir CM Daly GR Hembrecht S Keelan S Giblin K. Diagnostic accuracy of intraoperative methods for margin assessment in breast cancer surgery: A systematic review & meta-analysis. Breast. (2024) 76:103749. doi: 10.1016/j.breast.2024.103749, PMID: 38759577 PMC11127275

[49] Jin X Zhou YF Ma D Zhao S Lin CJ Xiao Y. Molecular classification of hormone receptor-positive HER2-negative breast cancer. Nat Genet. (2023) 55:1696–708. doi: 10.1038/s41588-023-01507-7, PMID: 37770634

[50] Chau OW Fakir H Lock M Dinniwell R Perera F Erickson A. Dosimetric planning comparison for left-sided breast cancer radiotherapy: the clinical feasibility of four-dimensional-computed tomography-based treatment planning optimization. Cureus. (2022) 14:e24777. doi: 10.7759/cureus.24777, PMID: 35673303 PMC9165918

[51] Ahn SH Kim E Kim C Cheon W Kim M Lee SB. Deep learning method for prediction of patient-specific dose distribution in breast cancer. Radiat Oncol. (2021) 16:154. doi: 10.1186/s13014-021-01864-9, PMID: 34404441 PMC8369791

[52] Stowe HB Andruska ND Reynoso F Thomas M Bergom C. Heart sparing radiotherapy techniques in breast cancer: A focus on deep inspiration breath hold. Breast Cancer (Dove Med Press). (2022) 14:175–86. doi: 10.2147/BCTT.S282799, PMID: 35899145 PMC9309321

[53] Wolf J Stoller S Lübke J Rothe T Serpa M Scholber J. Deep inspiration breath-hold radiation therapy in left-sided breast cancer patients: a single-institution retrospective dosimetric analysis of organs at risk doses. Strahlenther Onkol. (2023) 199:379–88. doi: 10.1007/s00066-022-01998-z, PMID: 36074138 PMC10033469

[54] Kefeli AU Diremsizoglu U Erdogan S Karabey AU Konuk AO Tirpanci B. Patient coaching for deep inspiration breath hold decreases set-up duration and left anterior descending artery dose for left-sided breast cancer radiotherapy. Support Care Cancer. (2025) 33:387. doi: 10.1007/s00520-025-09446-1, PMID: 40240656 PMC12003517

[55] Lalani N Alqarni S Jimenez RB. The potential of proton therapy for locally advanced breast cancer: clinical and technical considerations. Curr Oncol. (2023) 30:2869–78. doi: 10.3390/curroncol30030219, PMID: 36975432 PMC10047123

[56] Holt F Probert J Darby SC Haviland JS Coles CE Kirby AM. Proton beam therapy for early breast cancer: A systematic review and meta-analysis of clinical outcomes. Int J Radiat Oncol Biol Phys. (2023) 117:869–82. doi: 10.1016/j.ijrobp.2023.02.023, PMID: 36868521 PMC7615202

[57] Mutter RW Choi JI Jimenez RB Kirova YM Fagundes M Haffty BG. Proton therapy for breast cancer: A consensus statement from the particle therapy cooperative group breast cancer subcommittee. Int J Radiat Oncol Biol Phys. (2021) 111:337–59. doi: 10.1016/j.ijrobp.2021.05.110, PMID: 34048815 PMC8416711

[58] Taylor CW Wang Z Macaulay E Jagsi R Duane F Darby SC. Exposure of the heart in breast cancer radiation therapy: A systematic review of heart doses published during 2003 to 2013. Int J Radiat Oncol Biol Phys. (2015) 93:845–53. doi: 10.1016/j.ijrobp.2015.07.2292, PMID: 26530753

[59] Salim N Popodko A Tumanova K Stolbovoy A Lagkueva I Ragimov V. Cardiac dose in the treatment of synchronous bilateral breast cancer patients between three different radiotherapy techniques (VMAT, IMRT, and 3D CRT). Discov Oncol. (2023) 14:29. doi: 10.1007/s12672-023-00636-z, PMID: 36862205 PMC9981832

[60] Coon AB Dickler A Kirk MC Liao Y Shah AP Strauss JB. Tomotherapy and multifield intensity-modulated radiotherapy planning reduce cardiac doses in left-sided breast cancer patients with unfavorable cardiac anatomy. Int J Radiat Oncol Biol Phys. (2010) 78:104–10. doi: 10.1016/j.ijrobp.2009.07.1705, PMID: 20004529

[61] Zhang W Wang S Xing Y Luo X Wang R Yu F. Bioorthogonal SERS-bioluminescence dual-modal imaging for real-time tracking of triple-negative breast cancer metastasis. Acta Biomater. (2025) 197:431–443. doi: 10.1016/j.actbio.2025.03.019, PMID: 40101869

[62] Gunster JLB Schrijver AM van Duijnhoven FH Stokkel MPM Marijnen CAM Scholten AN. Impact of routine FDG-PET/CT on locoregional treatment decisions in breast cancer patients receiving preoperative systemic therapy. Breast. (2025) 81:104475. doi: 10.1016/j.breast.2025.104475, PMID: 40334384 PMC12138562

[63] Ng J Pennell R Formenti SC. The initial experience of MRI-guided precision prone breast irradiation with daily adaptive planning in treating early stage breast cancer patients. Front Oncol. (2022) 12:1048512. doi: 10.3389/fonc.2022.1048512, PMID: 36505797 PMC9728922

[64] Kim A Kalet AM Cao N Hippe DS Fang LC Young L. Effects of preparatory coaching and home practice for deep inspiration breath hold on cardiac dose for left breast radiation therapy. Clin Oncol (R Coll Radiol). (2018) 30:571–7. doi: 10.1016/j.clon.2018.04.009, PMID: 29773446

[65] Melemenidis S Viswanathan V Dutt S Kapadia N Lau B Soto LA. Effectiveness of FLASH vs. Conventional dose rate radiotherapy in a model of orthotopic, murine breast cancer. Cancers (Basel). (2025), 17. doi: 10.3390/cancers17071095, PMID: 40227580 PMC11988084

[66] Wang B Ting CY Lai CS Tsai YS. Bismuth pelvic X-ray shielding reduces radiation dose exposure in pediatric radiography. BioMed Res Int. (2021) 2021:9985714. doi: 10.1155/2021/9985714, PMID: 34671681 PMC8523245

